# The eye is a common site of granulomatosis with polyangiitis. A collaborative study

**DOI:** 10.1186/s12886-022-02743-x

**Published:** 2023-01-18

**Authors:** Rosanna Dammacco, Jyotirmay Biswas, Amanda Mohanan-Earatt, Walter Lisch, Francesco Alfredo Zito, Giuseppe Rubini, Carlo Manno, Sebastiano Cicco, Giovanni Alessio, Franco Dammacco

**Affiliations:** 1 Department of Ophthalmology and Neuroscience, University of Bari “Aldo Moro”, Medical School, Bari, Italy; 2 Department of Uveitis and Ocular Pathology, Sankara Nethralaya, Chennai, India; 3 Department of Ophthalmology, Johannes Gutenberg University Mainz, Mainz, Germany; 4 Pathology Department, IRCCS-Istituto Tumori ‘Giovanni Paolo II’, Bari, Italy; 5 Nuclear Medicine Unit, University of Bari Medical School, Bari, Italy; 6 Department of Emergency and Organ Transplantation, Nephrology, Dialysis and Transplant Unit, University of Bari “Aldo Moro”, Bari, Italy; 7 Department of Biomedical Sciences and Human Oncology, University of Bari “Aldo Moro”, Medical School, Bari, Italy

**Keywords:** Granulomatosis with polyangiitis, Episcleritis, Scleritis, Orbital inflammatory disease, ANCA-associated vasculitis, Rituximab

## Abstract

**Background:**

Ocular manifestations of granulomatosis with polyangiitis (GPA) have been reported in a limited number of studies and with largely variable frequency. Here we report on the clinical, diagnostic, and therapeutic features of a cohort of 63 GPA patients, with particular regard to 22 of them with ophthalmic involvement (35%).

**Methods:**

Clinical manifestations, results of immunological findings, histopathological pictures, imaging data, Birmingham Vasculitis Activity Score, therapeutic regimens, and outcomes were retrospectively analyzed. At diagnosis, in addition to a structured clinical assessment, all patients underwent a comprehensive ophthalmologic examination.

**Results:**

The most frequently involved organs were kidneys, lungs, ear/nose/throat, and eyes. Ocular manifestations were bilateral in 32%. The three most commonly diagnosed ophthalmologic manifestations were scleritis (36%), retro-orbital pseudotumor or orbital mass (23%), and episcleritis (13%). Ocular and systemic involvement were simultaneously present at onset in 41% of the patients; systemic involvement was followed by ocular lesions in 36%; ocular inflammation was followed by systemic manifestations in 18%; and an orbital mass in the absence of systemic disease characterized 5%. Glucocorticoids plus cyclophosphamide and glucocorticoids plus rituximab were the combined therapies most frequently employed during remission induction and remission maintenance, respectively. Persistent ophthalmologic and extra-ocular remissions were achieved in 77 and 64% of the patients, respectively. One to three systemic relapses were diagnosed in 7 patients (31.8%). At the last follow-up, a visual outcome 20/40 or better in 31 (70%) of 44 eyes was determined.

**Conclusions:**

The eye was involved in over one third of our patients with GPA. Increased awareness, early diagnosis, and multi-specialty collaboration are critical in achieving a favorable outcome of GPA.

## Background

Granulomatosis with polyangiitis (GPA), formerly called Wegener’s granulomatosis, is a rare and often severe multiorgan vasculitis characterized by a necrotizing granulomatous inflammation of the small to medium-sized vessels, leading to endothelial damage and tissue injury. Together with microscopic polyangiitis and eosinophilic granulomatosis with polyangiitis (previously known as Churg-Strauss syndrome), GPA belongs to the group of anti-neutrophil cytoplasmic antibody (ANCA)-associated vasculitides (AAV) [1–3]. While there are substantial differences in the incidence rate of GPA in Europe and Asia and as a function of latitude, the overall incidence rate of the disease is 0.4–11.9 cases/million person-years, with a prevalence of 2.3–146.0 cases/million persons [2]. Epidemiological studies, however, have documented a progressive increase in GPA, reflecting a wider awareness of the disease and the increased availability of reliable imaging techniques. In a population-based cohort study carried out in Denmark, the median annual incidence rate was 20.5/million persons, with an increase in the point prevalence from 64/million in 1995 to 277/million in 2015 [4]. GPA can develop in both sexes but there is a slight female preponderance. The peak age at onset is 45–65 years, but the disease can be detected at any age, including in the pediatric population [5].

The immunologic hallmark of GPA is the almost unfailing occurrence of ANCAs, which can be divided into c-ANCAs and p-ANCAs, depending on whether the immunofluorescent staining pattern is cytoplasmic or perinuclear. The cytoplasmic autoantigen targets of c-ANCAs and p-ANCAs are proteinase-3 (PR3, a serine protease expressed mainly in neutrophil granulocytes) and myeloperoxidase (MPO, an enzyme stored in the azurophilic granules of neutrophils and monocytes), respectively [6]. Anti-PR3 ANCAs can be detected in ~ 70–75% of GPA patients, with variations across different ethnic groups and depending on whether the disease is in the active or quiescent phase; anti-MPO ANCAs occur in ~ 15–20% of GPA patients. However, up to 20% of patients may be both anti-PR3 and anti-MPO negative [2], especially those with less severe disease or with localized granulomatous disease of the upper or lower respiratory tract. IgG antibodies directed against long pentraxin-3 (PTX3), a recognition receptor produced by various cell types, have been detected in nearly 30% of GPA patients, half of whom are c- and p-ANCA negative. Anti-PTX3 antibodies can therefore be considered a novel biomarker of AAV [7, 8].

The clinical features and presentations of GPA patients at diagnosis are remarkably heterogeneous, ranging from a lack of symptoms to single-organ involvement with minimally symptomatic disease to organ- or life-threatening manifestations. Frequently, the onset of GPA is characterized by the presence of constitutional symptoms that by weeks or a few months precede the clinical presentation. Although the granulomatous and vasculitic lesions can affect any organ system or tissue, upper and lower respiratory tract injury and rapidly progressive glomerulonephritis are the most common [2, 3, 6, 9], but why the lung and kidney are preferentially affected is unknown.

Ocular manifestations have been recorded with frequencies ranging from 13% [10] to 60% [11] and are either the sole presenting feature (~ 15% of patients) or, as is more often the case, appear at variable time during the course of the disease [12, 13]. Although the most common ophthalmologic diagnoses are scleritis, episcleritis, and orbital masses, every structure of the eye can be affected. Moreover, involvement is bilateral in up to 58% of patients [12], with a permanent visual loss reported in 5–10% [11].

In the following, we summarize the clinical, diagnostic, and therapeutic features of GPA in a longitudinal cohort of 22 patients with visual system involvement who were diagnosed and followed-up at tertiary referral centers with specific experience in ophthalmology and clinical immunology.

## Materials and methods

This was a retrospective, cross-sectional, observational study of 63 consecutive Caucasian GPA patients, whose medical records were obtained from a computerized database. All patients had been admitted from May 2005 to December 2019 to the Department of Internal Medicine of the University of Bari, Italy, that is a tertiary referral center for all AAV, and then examined at the University’s Department of Ophthalmology and Neuroscience with the aim of describing those with ocular involvement. The ethical approval for the study was given by the Ethics Committee of the University of Bari Medical School and all procedures were performed in accordance with relevant guidelines.

At diagnosis, all patients underwent a comprehensive ophthalmologic examination that included best-corrected visual acuity (BCVA) measured on the Snellen chart, intraocular pressure (IOP) by applanation tonometry, a complete biomicroscopic assessment, ocular motility, visual field testing, fluorescein or indocyanine green angiography, and optical coherence tomography. The patients were grouped according to their BCVA grade as follows: grade I, 20/25 or better; grade II, 20/30 to 20/40; grade III, 20/50 to 20/160; grade IV, 20/200 or worse [14, 15]. All tests were performed or repeated as required at any time during the course of the disease.

All patients in this cohort met the 1990 American College of Rheumatology classification criteria for GPA [16] and the revised nomenclature established by the Chapel Hill international consensus conference [1]. A structured clinical examination together with a careful appraisal of both the immunologic and histopathologic findings and the available imaging data were performed for each patient at presentation and during follow-up. In addition to routine investigations, chest X-ray, and abdominal ultrasound, the ‘ANCA Profile’ kit (Euroimmun, Lübeck, Germany) was used for ANCA serology, with few exceptions. The results of positive indirect immunofluorescence, performed to determine the occurrence and the c- or p-pattern of ANCAs, were always confirmed with a monospecific anti-PR3 and anti-MPO enzyme-linked immunosorbent assay (ELISA). The combined procedure enhanced both sensitivity and specificity to over 90%. Computed tomography (CT) and ^18^F-fluorodeoxyglucose positron emission tomography/computed tomography (FDG-PET/CT) of the chest and other body districts were carried out as warranted by the clinical features [17]. Biopsy of the involved tissue(s) was performed in 31 of the 63 patients (49.2%), including 9 of the 22 patients (40.9%) with ophthalmologic symptoms. Detection of the typical histopathologic triad of granulomatous inflammation, geographic necrosis, and necrotizing small vessel vasculitis was considered diagnostic of GPA [18].

All definitions of disease activity followed the recommendations developed by the European League Against Rheumatism (EULAR) [19]. Accordingly, response to treatment was defined as a ≥ 50% reduction in the disease activity score, and remission as the absence of disease activity. However, since GPA tends to flare or its activity may fluctuate (grumbling disease), remission was qualified by its duration. Relapse was defined as the re-occurrence or new onset of disease activity ascribable to active inflammation and recorded as either major or minor, depending on whether it was potentially organ- or life-threatening. Refractory disease was defined as the failure to achieve remission following a standard induction treatment [19].

Five levels of disease severity, and thus treatment protocols of variable strength, were adopted according to the criteria proposed by the European Vasculitis Study Group [20]: 1) localized: upper and/or lower respiratory tract disease in the absence of other systemic involvement or constitutional symptoms; 2) early systemic: any organ, without organ-threatening or life-threatening disease; 3) generalized: renal or other organ-threatening disease, serum creatinine < 5.6 mg/dL; 4) severe: renal or other vital organ failure, serum creatinine > 5.6 mg/dL; 5) refractory: progressive disease unresponsive to glucocorticoids (GC) and cyclophosphamide (CYC). Disease category and the corresponding treatment decisions were established at diagnosis and reappraised during subsequent examinations. The median follow-up was 51 months (range 21–84 months) after the onset of ocular involvement. The Birmingham Vasculitis Activity Score version 3 (BVASv3) was employed as a validated tool in the assessment of disease activity. Active vasculitis was defined as BVASv3 ≥ 1 and inactive disease as BVASv3 = 0 [21, 22].

Categorical data are reported as numbers and corresponding percentages. Group data were compared using Fisher’s exact test and means using an unpaired Student *t* test. A *p* value < 0.05 was considered statistically significant.

## Results

**Fig.1:**
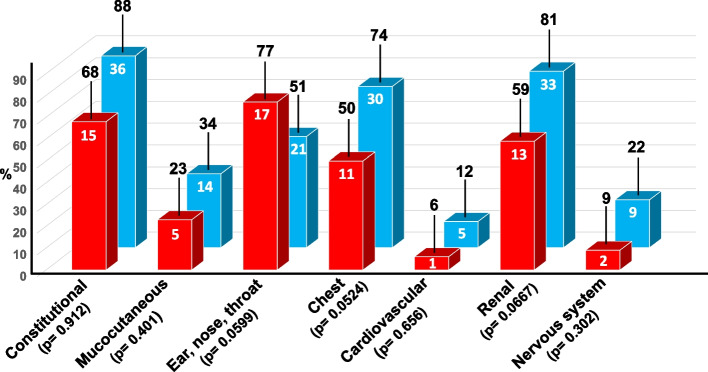
A comparative analysis of the clinical manifestations recorded at diagnosis in the 22 patients with granulomatosis with polyangiitis (GPA) and ophthalmic involvement (red bars, group A), and in the 41 GPA patients without ophthalmic involvement (blue bars, group B). The differences between the two groups are not significant. The white numbers inside the bars indicate the number of patients, whereas the black numbers on top of each bar indicate the corresponding percentages

Our cohort of 63 GPA patients consisted of 36 females (57.1%) and 27 males (42.8%), with an average age at presentation of 53.5 years (range 29–77). Ophthalmic manifestations were detected in 22 patients (34.9%), including 13 females (59.1%) and 9 males (40,9%), with a mean age of 55 years (range, 35–73 years). These 22 patients with visual system involvement (group A) were compared with those whose eyes remained unaffected throughout the follow-up period (group B). As shown in Fig. 1, with the exception of the ENT system, involvement of the different organs was consistently higher in group B (blue bars) than in group A (red bars), but the differences between the two groups were not statistically significant.

As reported in major clinical studies [10, 23–26], constitutional symptoms, such as irregular fever ≥38 °C, weight loss, fatigue, and arthro-myalgias, as well as mucocutaneous manifestations, including purpuric eruptions and oral as well as genital ulcers, were present in variable combinations in the large majority of our patients, either at presentation or during the first few months after disease onset. These general and non-specific signs and symptoms often heralded the onset of those recognized as more typical and related, in decreasing order of frequency, to involvement of the kidneys, lungs, ear/nose/throat (ENT), eyes, and nervous and cardiovascular systems.

Table 1 provides an overview of the ocular and extraocular manifestations, ANCA pattern, and disease activity of the 22 patients. Nine patients (40.9%) had both ocular and systemic involvement at onset; 8 patients (36.3%) initially had systemic involvement and later developed an ocular lesion; in 4 patients (18.2%) ocular inflammation was followed by systemic manifestations; and one patient (4.5%) had an isolated orbital mass in the right eye and mild constitutional symptoms but did not develop systemic disease at any time during follow-up. Ocular involvement was unilateral in 15 patients (68.2%) and bilateral in 7 patients (31.8%). The IOP at diagnosis was normal in 41 eyes whereas a mild increase but no development of glaucoma was determined in 3 eyes of 3 patients diagnosed with necrotizing scleritis, with scleral melt detected in one and orbital inflammatory pseudotumor in the other two.

**Table 1:** Demographic features, ocular and extra-ocular manifestations at disease onset or developed during follow-up, ANCA pattern and disease activity in 22 patients with granulomatosis with polyangiitis

Pt. No.	Age, Sex	BCVA OD OS	IOP OD OS	Ophthalmologic Diagnosis	Extraocular Organ or System Involvement	ANCA Pattern of Positivity	BVASv3
1	48, M	20/60 20/40	13 14	Peripheral ulcerative keratitis OD	Arthralgia/arthritis, paranasal sinusitis, bilateral lung infiltrates, proteinuria > 1.5 g/day	c-ANCA	NA/NP
2	59, F	20/60 20/40	15 14	Diffuse episcleritis OU	Fever, weight loss, fatigue, increased serum creatinine levels	c-ANCA	9
3	54, F	20/60 20/40	13 14	Diffuse episcleritis OD and conjunctivitis OS	Constitutional, sinusitis, proteinuria, hematuria, hypertension, congestive heart failure	c-ANCA	23
4	68, F	20/40 20/50	13 14	Nodular episcleritis and conjunctivitis OS	Paranasal sinusitis, proteinuria, hypertension	c-ANCA	13
5	46, F	20/40 20/60	14 18	Unspecified scleritis OS	General and mucocutaneous symptoms, paranasal sinusitis, urinary abnormalities, hypertension	c-ANCA	23
6	60, M	20/40 20/40	13 14	Unspecified scleritis OU	General, nasal granulomata, torpid genital ulcers, mild proteinuria	c-ANCA	14
7	35, M	20/40 20/40	18 14	Diffuse anterior scleritis OU	Mild constitutional symptoms, sinusitis, urinary protein output > 1 g/24 hr	c-ANCA	9
8	55, F	20/40 20/40	13 14	Diffuse anterior scleritis OS with peripheral ulcerative keratitis OD	Serotine fever, myalgias, conductive hearing loss, right lung with initial cavitating infiltration	NA/NP	13
9	51, M	20/60 20/40	16 14	Necrotizing scleritis OD	Constitutional symptoms, mouth ulcers, bloody nasal discharge, bilateral pleural effusion	p-ANCA	18
10	41, F	20/160 20/160	13 20	Necrotizing scleritis OU	Pulmonary nodules, serum creatinine > 30% of ULN, nasal crusts, mucocutaneous and general symptoms	NA/NP	29
11	65, F	20/40 20/80	15 19	Necrotizing scleritis OS	General, bilateral cavitating pulmonary lesions, saddle nose deformity	NA/NP	18
12	64, F	20/200 20/40	23 16	Necrotizing scleritis, scleral melt and uveal prolapse OD	Patchy ground-glass opacities in both lungs, nasal ulcers and granulomata, constitutional symptoms	NA/NP	24
13	71, F	20/40 20/80	14 18	Circumscribed scleromalacia OS	Necrotizing glomerulonephritis, mononeuritis multiplex	c-ANCA	27
14	46, M	20/200 20/40	13 14	Orbital mass OD	Irregular fever, arthromyalgias	c-ANCA	9
15	66, M	20/140 20/40	13 14	Retro-orbital pseudotumor with proptosis OD	Bloody-purulent nasal discharge, purpura of the legs, pleural effusion	NA/NP	16
16	58, M	20/40 20/120	15 24	Left orbital inflammatory pseudotumor	Fever, weight loss and arthralgia, recurrent bloody nasal discharge with nasal septum perforation	c-ANCA	15
17	59, F	20/40 20/40	13 14	Right orbital mass with dacriocystitis	Nasal granulomata, persistent proteinuria in the nephrotic range and hematuria	c-ANCA	20
18	73, F	20/40 HM	18 26	Left retro-orbital Inflammatory pseudotumor with diplopia	Constitutional, purpuric eruptions, paranasal sinusitis, bilateral pulmonary nodules, focal glomerulonephritis	c-ANCA	NA/NP
19	60, F	20/160 20/120	13 14	Orbital inflammatory disease OU	Rhinosinusitis, rapidly progressive glomerulonephritis	ANCA-neg → pos	18
20	43, M	CF 20/40	13 14	Compressive neuropathy OD	Consolidated nodules in both lungs, biopsy-shown crescentic glomerulonephritis	c-ANCA	NA/NP
21	50, F	20/40 CF	13 14	Optic perineuritis with compressive neuropathy OS	Ground-glass opacities in the left lung, radiological signs of pulmonary hemorrhages in the right lung	c-ANCA	12
22	47, M	20/40 20/160	13 14	Retinal vasculitis with blurring vision OS	Patchy ground-glass opacities in the right lung, sensory peripheral neuropathy	c-ANCA	16

*BCVA* best-corrected visual acuity, *BVASv3* Birmingham Vasculitis Activity Score version 3 (Mukhtyar C et al.^21^), CF counting fingers, *HM* hand motion, *IOP* intraocular pressure (normal values 10–21 mmHg), *NA/NP* not available or not performed, *OD* right eye, *OS* left eye, *OU* both eyes, *ULN* upper limit of normal

**Fig. 2:**
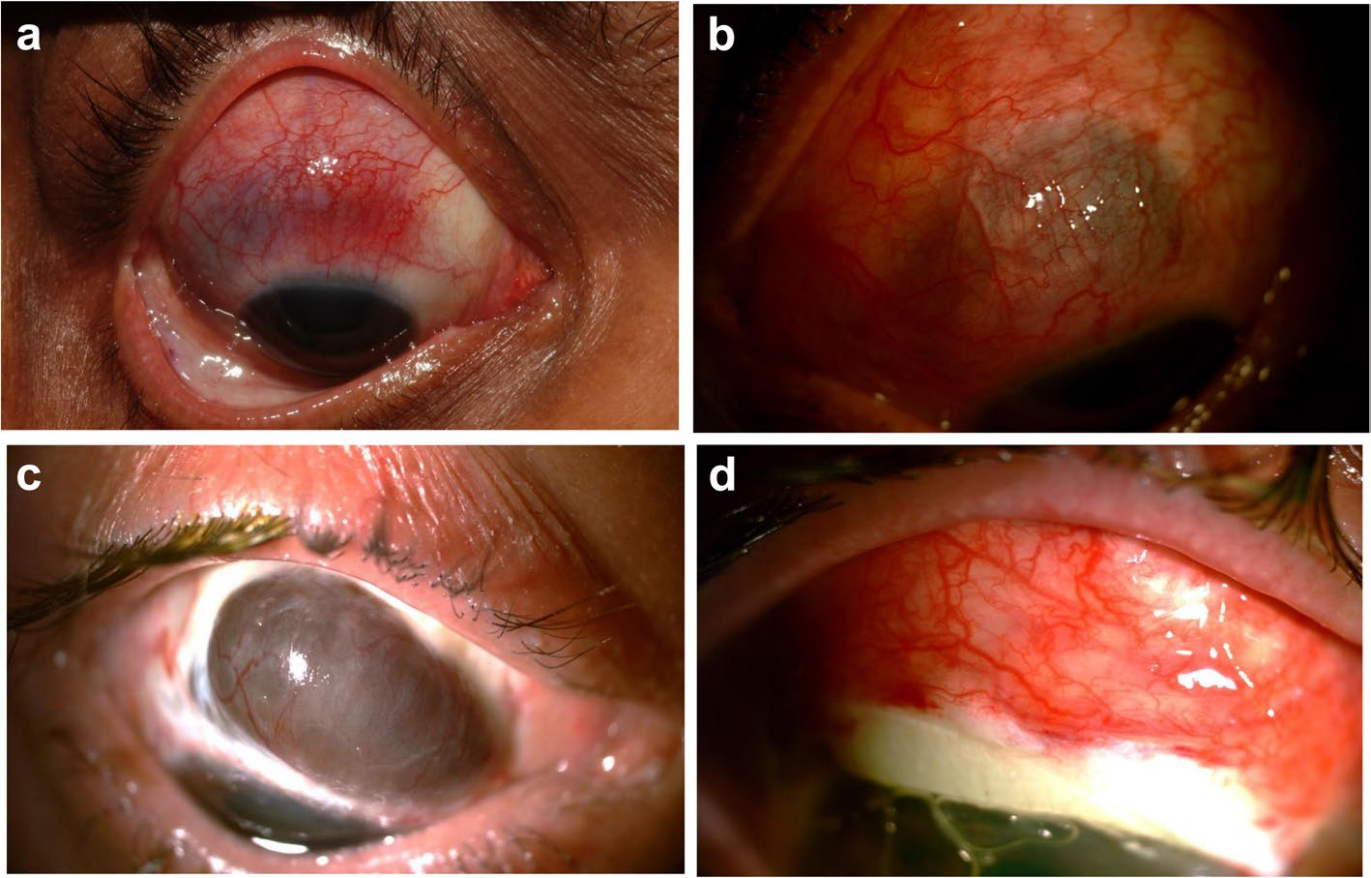
**a**) Necrotizing scleritis of the right eye at presentation in acute phase. The patient (number 9 of Table 1), who was c-ANCA-negative and p-ANCA-positive, was treated with a combination of GC plus CYC, which resulted in total regression of the scleritis and a substantial reduction of the pulmonary nodules. **b**) Left eye in a woman (number 10 of Table 1) at presentation with active necrotizing scleritis OU and multi-organ GPA. She was given six infusions of CYC and four infusions of RTX intravenously. Her scleritis, which appeared while she was on maintenance therapy with MMF plus GC, resolved with the addition of CYC. **c**), **d**) Active necrotizing scleritis of the right eye in a woman (number 11 of Table 1) with severe lung involvement, showing scleral melt and uveal prolapse superiorly (2c). Two doses of RTX were administered to control inflammation, followed by a scleral patch graft (2d) and maintenance therapy consisting of azathioprine, MMF, and GC. At the patient’s last follow-up, her scleritis had resolved, the graft had integrated, and her eye condition was considered stable

The frequency of the various ophthalmic manifestations is summarized in Table 2. Peripheral ulcerative keratitis was diagnosed in 2 patients (9.1%), as the sole ophthalmologic abnormality in one of them and associated with diffuse anterior scleritis in the other. Three patients (13.6%) had episcleritis, associated with conjunctivitis in 2 of them. The most frequent ophthalmologic abnormality was scleritis (Fig. 2), diagnosed in 9 patients (40.9%) as follows: unspecified in 2 patients; diffuse anterior in 2 patients, including 1 in whom scleritis in one eye was associated with peripheral ulcerative keratitis in the other eye; necrotizing in 4 patients, one of whom also had scleral melt and uveal prolapse; and manifesting as circumscribed scleromalacia in 1 patient.

**Table 2:** Ophthalmic manifestations in 22 patients with GPA

Manifestation^a^	No. of patients (%)
Peripheral ulcerative keratitis	2 (9.1)
Episcleritis	3 (13.6)
Nodular	2
Diffuse	1
Scleritis	9 (40.9)
Unspecified	2
Diffuse anterior	2
Necrotizing	4
Necrotizing with scleral melt	1
Uveal prolapse	1 (4.5)
Circumscribed scleromalacia	1 (4.5)
Orbital mass	2 (9.1)
Retro-orbital pseudotumor	3 (13.6)
Compressive neuropathy	2 (9.1)
Retinal vasculitis	1 (4.5)
Laterality of eye involvement	
Unilateral	15 (68.2)
Bilateral	7 (31.8)

^a^Three patients had more than one ocular manifestation

Orbital disease was recorded in 6 patients (27.3%), including 3 patients in whom it was associated with proptosis, dacriocystitis, and diplopia, respectively. Another patient with bilateral orbital inflammatory disease was initially diagnosed with IgG4-related ophthalmic disease [27, 28], but she was later reassessed, as immuno-histochemical staining of her orbital biopsy specimens showed very few IgG4-positive plasma cells and a low IgG4/IgG plasma cell ratio, poor collagenous fibrosis, the absence of obliterative phlebitis or eosinophil infiltration, sparing of the lacrimal gland, and a normal serum IgG4 concentration. She was ANCA-negative at presentation but became strongly positive 3 months later. Compressive neuropathy was detected in 2 patients (9.1%) and retinal vasculitis in 1 patient (4.5%). None of our 22 GPA patients with ophthalmic complications were diagnosed with drainage system involvement.

**Fig. 3:**
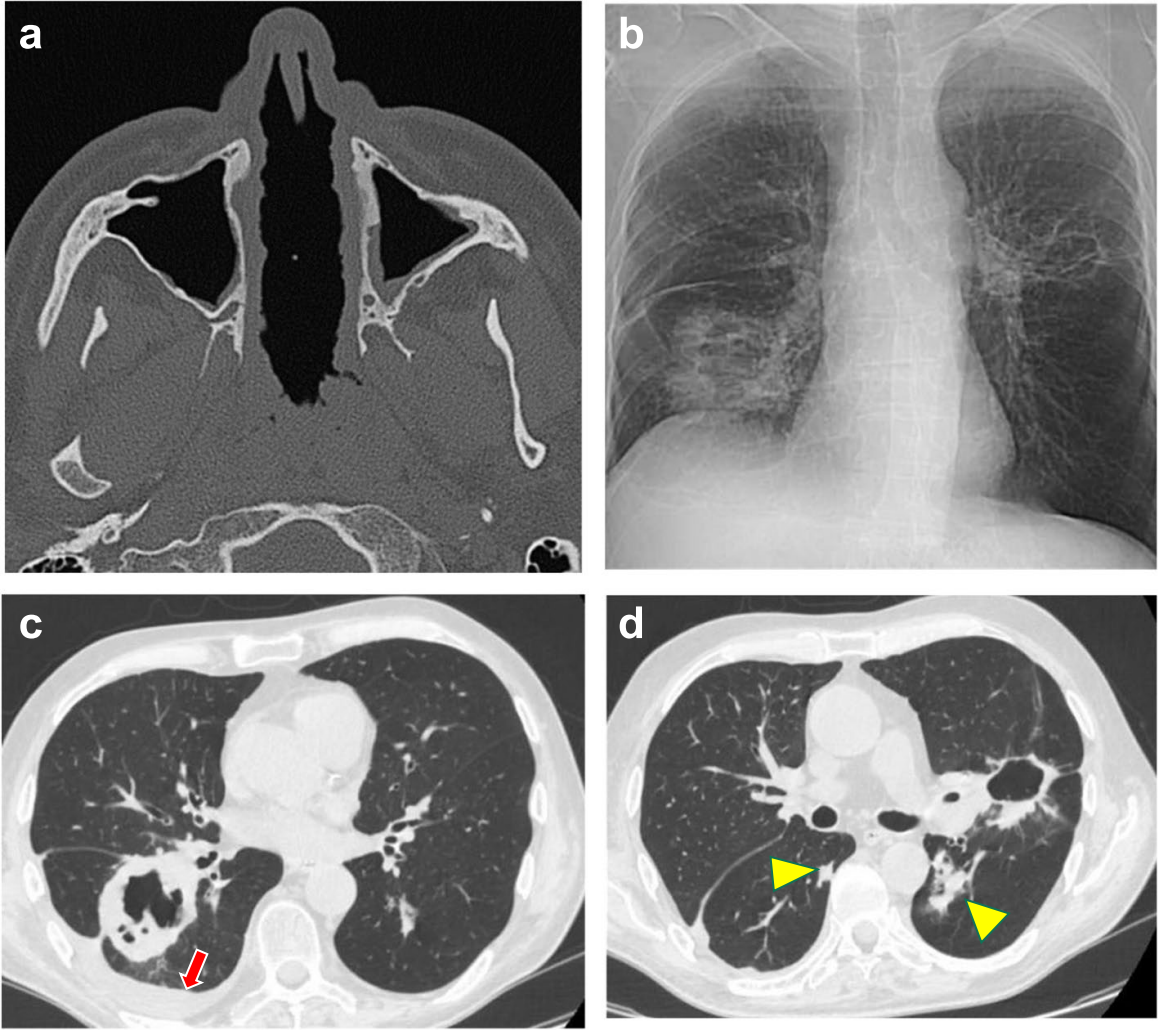
**a**) Necrotizing perforation of the nasal septum (patient 16 of Table 1). Axial CT demonstrates the nearly complete absence of the nasal septum. The mucosa appears thickened and nodular. **b**) Chest X-ray (patient 11 of Table 1), showing a large cavitary nodule with uneven and thickened walls in the right basal area and interlobar imbibition. An additional peri-hilar cavitary lesion with a clean, thin wall is also recognizable in the left lung. **c**) Chest CT confirms the presence of a cavitary granulomatous lesion with an anfractuous inner wall close to the right hilum and pulmonary fissure. The red arrow points to a modest layer of pleural effusion. **d**) The cavitary lesion close to the left hilum has a thinner, almost regular wall. Additional nodules of variable size are present in both lungs (yellow arrowheads)

**Fig. 4:**
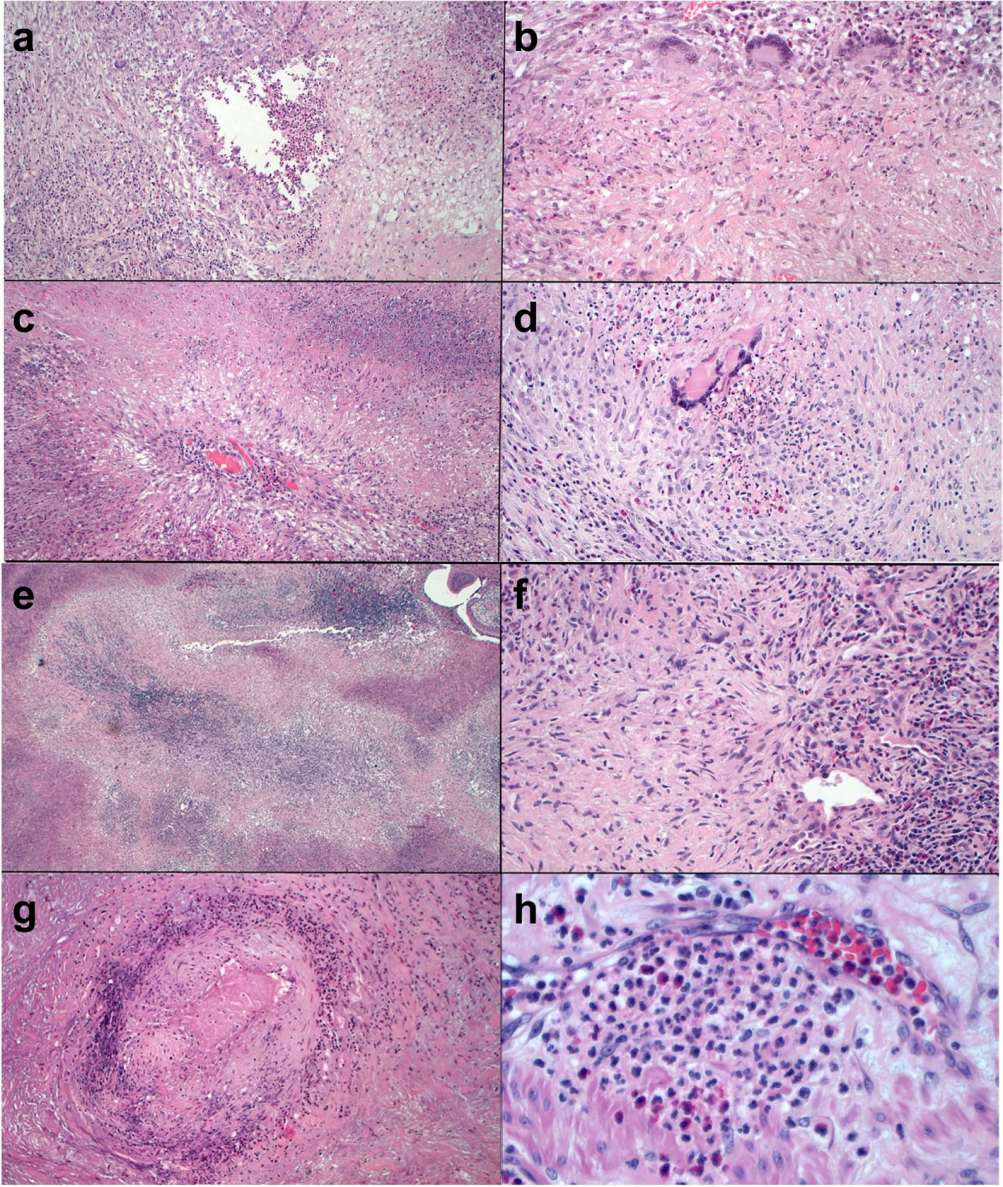
Granulomas and vasculitis in surgical lung biopsies from patient 1 (**a**-**d**) and patient 22 (e-h) of Table 1: **a**) This necrotizing granuloma has a central area with eosinophilic inflammation surrounded by chronic inflammation with multinucleated giant cells (hematoxylin-eosin [H&E], × 10). **b**) Multinucleated giant cells and lympho-plasmocytic cells surround a fibro-histiocytic central area with focal necrosis (H&E, × 20). **c**) This granuloma consists of a cartwheel-shaped arrangement of palisading histiocytes surrounding a central arteriole in a necrotic background (H&E, × 10). d) A granuloma with central acute inflammation (H&E, × 20). **e**) Large zones of basophilic necrosis with an irregular border give the appearance of geographic necrosis (H&E, × 4). **f**) Nodular scar with central multinucleated giant cells and adjacent vasculitis (H&E, × 20). **g**) Necrotizing arteritis. The wall of this arteriole shows marked inflammation and central fibrinoid necrosis (H&E, × 10). **h**) Neutrophilic capillaritis: the inflammation infiltrates the capillary wall with spilling over into the hematic space (H&E, × 40)

Upper or/and lower airway symptoms included nasal, sinus, and pulmonary abnormalities, occurring in isolation or in variable combinations. The ENT system was involved in 17 of the 22 patients (77%); the clinical features included bloody nasal discharge, nasal crusts and/or ulcers, and sinusitis. In 2 patients, the inflammatory and granulomatous processes led to perforation of the nasal septum (Fig. 3a) and consequent saddle nose deformity. 11 patients (50%) presented with pulmonary symptoms of variable severity, ranging from persistent cough and hemoptysis to cavitating pulmonary lesions (Fig. 3b–d) and patchy or diffuse ground-glass opacities. Histopathologic examination of surgically achieved lung tissue showed granulomatous inflammation, stenotic involvement of the vascular lumen, infiltration of immune cells surrounding the vessels and disruption of the vascular wall (Fig. 4).

**Fig. 5:**
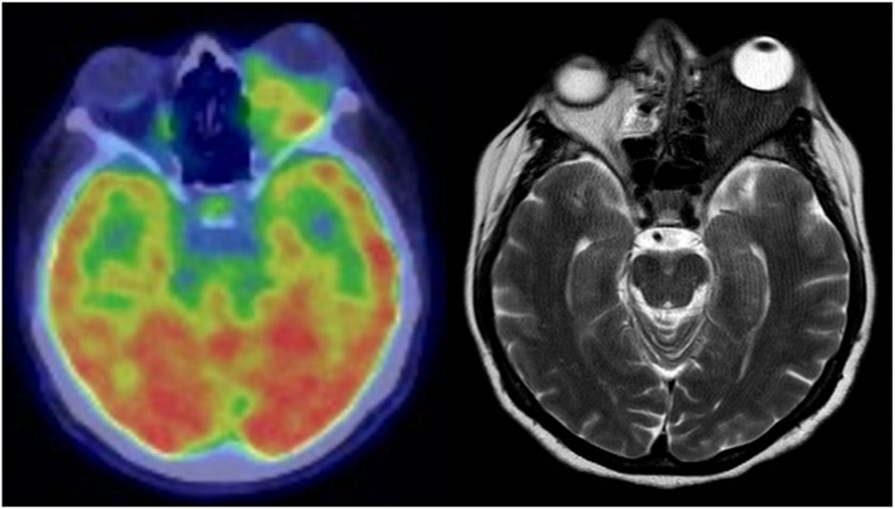
**a**) ^18F^FDG-PET/CT (patient 18 of Table 1) shows an abnormally increased FDG uptake and **b**) MR T2-weighted in transaxial section reveals a large mass at the level of the left orbital cavity and in the retrobulbar area that caused marked proptosis

FDG-PET/CT, performed in 7 patients, was of particular diagnostic utility in one patient with left retro-orbital inflammatory pseudotumor (Fig. 5) and in another who had multiple nodular subcutaneous lesions of the chest and abdominal wall, the largest of which were localized to the paravertebral lumbar area and both buttocks.

**Fig. 6:**
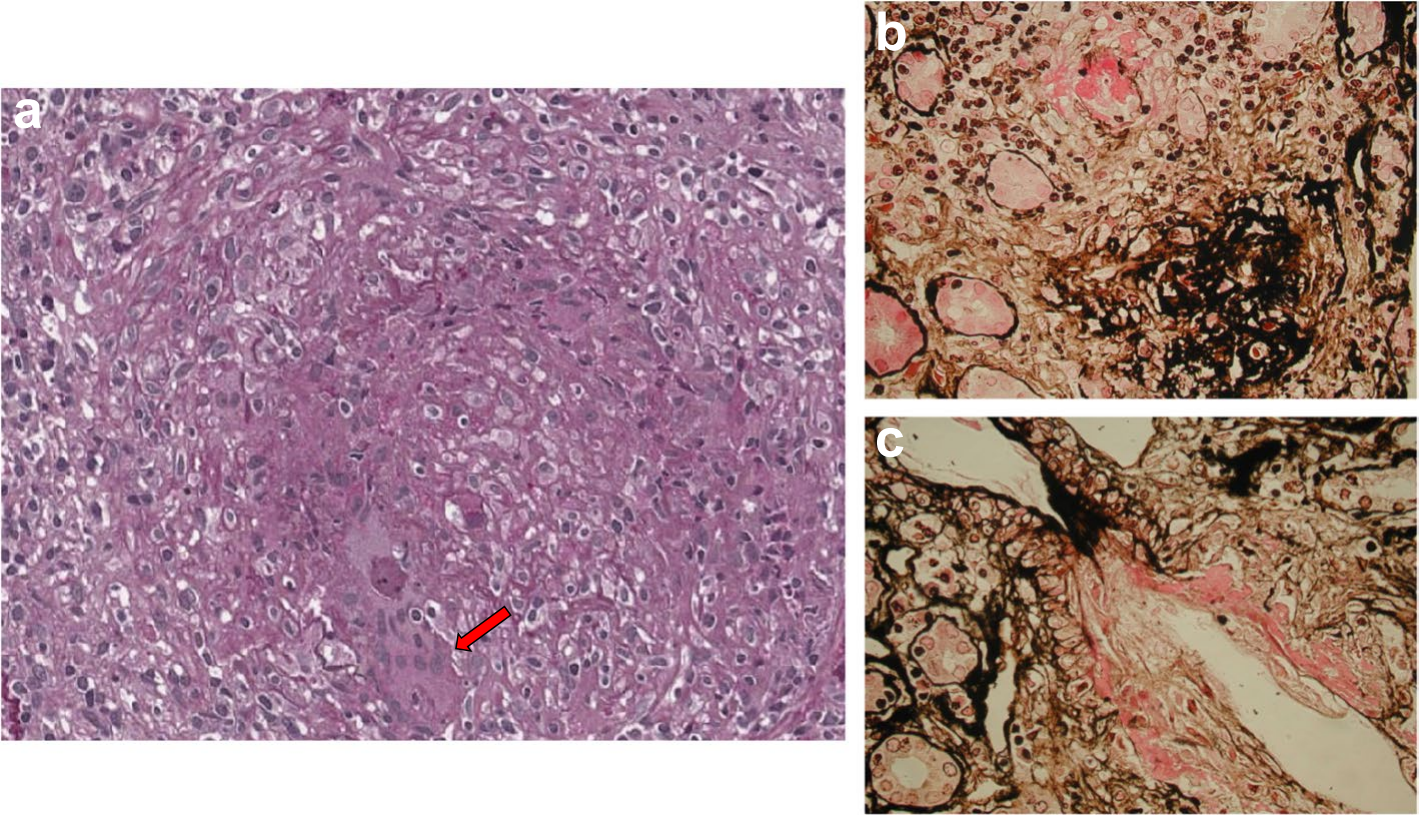
Renal biopsy from patient 20 of Table 1: **a**) The interlobular artery is extensively affected by a granulomatous necrotizing inflammatory process. The vessel wall is poorly recognizable due to the presence of wide areas of necrosis and leukocyte infiltration. Multinuclear giant cells can be seen in the infiltrate (arrow) (periodic acid Schiff, × 400). **b**) A small artery with fibrinoid necrosis of the wall and perivascular lympho-monocyte infiltration (methenamine silver, × 400). **c**) Fibrinoid necrosis of the wall of a small artery (methenamine silver, × 400)

Renal findings, detected in 13 patients (59%), included active urinary sediment, mild to nephrotic-range proteinuria, hematuria, increased serum creatinine levels, but also focal, rapidly progressive, or necrotizing glomerulonephritis that mostly developed during follow-up rather than occurring at presentation (Fig. 6a-c). Cardiac involvement, clinically manifesting as congestive heart failure, was diagnosed in one patient (4.5%). Nervous system involvement was detected in 2 patients (9%) diagnosed with mononeuritis multiplex and sensory peripheral neuropathy, respectively.

Since our cohort was assembled over a time frame of ~ 15 years, the drugs employed, their optimal doses, and their combinations were not the same for all patients (Table 3). Local GC therapy was initially restricted to patients with minor ocular manifestations, such as conjunctivitis and episcleritis, but systemic GC and immunosuppressive therapy were administered to these same patients when the initial response was poor and to all other patients with potential sight-threatening features, such as scleritis, peripheral ulcerative keratitis, and retinal vasculitis. None of the patients were treated with trimethoprim-sulfamethoxazole monotherapy as induction or maintenance therapy, although a dose of 2 × 960 mg/day is considered safe in patients with localized, non-severe disease and in the prevention of relapse [29].

**Table 3:** Therapeutic procedures, length of follow-up, BCVA, and outcomes in 22 patients with GPA and ocular manifestations

**TREATMENT** Drug combinations: • ivGC + ivCYC • oGC + oCYC • oGC + scMTX • oGC + oMMF • oGC + ivRTX	**REMISSION INDUCTION** No. of patients (%) • 2 (9.1) • 9 (40.9) • 3 (13.6) • 3 (13.6) • 5 (22.7)	**REMISSION MAINTENANCE** No. of patients (%) • 0 • 4 (18.2) • 2 (9.1) • 2 (9.1) • 14 (63.6)
**FOLLOW-UP**	**MEDIAN** • 51 months	**RANGE** • 21–84 months
**BCVA** • Grade I (20/25 or better) • Grade II (20/30 to 20/40) • Grade III (20/50 to 20/160) • Grade IV (20/200 or worse)	**AT DISEASE ONSET** No. of patients (%) • OD 0 - OS 0 • OD 11 (50) – OS 12 (54.5) • OD 8 (36.4) – OS 8 (36.4) • OD 3 (13.6) – OS 2 (9.1)	**AT LAST FOLLOW-UP** No. of patients (%) • OD 3 (13.6)- OS 2 (9.1) • OD 12 (54.5) – OS 14 (63.6) • OD 4 (18.2) – OS 4 (18.2) • OD 3 (13.6) – OS 2 (9.1)
**OUTCOMES**	**OPHTHALMOLOGIC OUTCOME** No. of patients (%) • Persistent remission: 17 (77.3) • Patients who relapsed 1 to 3 times: 5 (22.7): ° Necrotizing scleritis: 2 (9.1) ° Ulcerative keratitis: 1 (4.5) ° Orbital disease: 1 (4.5) ° Optic neuropathy: 1 (4.5)	**SYSTEMIC OUTCOME** No. of patients (%) • Persistent remission: 14 (63,6) • Patients who relapsed 1 to 3 times: 7 (31.8): ° Ear, nose, throat: 3 (13.6) ° Pulmonary: 3 (13.6) ° Renal: 1 (4.5) • Opportunistic infections: 3 (13.6) • Bacterial infections: 4 (18.2) • Death: 3 (13.6) due to: ° Generalized sepsis: 1 (4.5) ° Pneumonia: 1 (4.5) ° Colon carcinoma: 1 (4.5)

*BCVA* best-corrected visual acuity, *CYC* cyclophosphamide, *GC* glucocorticoids, *GPA* granulomatosis with polyangiitis, *iv* intravenous, *MMF* mycophenolate mofetil, *MTX* methotrexate, o oral, *RTX* rituximab, sc subcutaneous

To achieve remission induction, a conventional daily treatment with oral GC (0.5–1 mg/kg body weight) and oral CYC (2 mg/kg body weight) was given to 9 patients, and intravenous pulse methylprednisolone (usually 1–3 g) combined with intravenous pulse CYC (15–20 mg/kg body weight every 3 weeks) to 2 patients with organ- or life-threatening disease. Mesna (2-mercaptoethane sulfonate sodium) was added to prevent hemorrhagic cystitis from CYC, with patients simultaneously receiving generous hydration. Five patients (including patients 7, 10, and 22 in Table 1, for whom the preservation of fertility was prioritized) received the anti-CD20 monoclonal antibody rituximab (RTX: intravenous infusions of 375 mg/m^2^ weekly for a total of four doses) instead of CYC as the primary drug for remission induction, combined with oral GC. In 2 patients with an orbital mass and in 3 patients with retro-orbital pseudotumor, the disease was unresponsive to CYC. A switch to GC plus RTX resulted in better efficacy, but in one of the patients orbital decompression and the debulking of granulomas became necessary to relieve the compressive optic neuropathy. Three patients were treated with subcutaneous injections of methotrexate (MTX, 0.3 mg/kg body weight per week) in conjunction with GC and 3 patients received a combination of GC plus oral mycophenolate mofetil (MMF, 1 g twice daily). No patient underwent plasma exchange or received high-dose intravenous immunoglobulins, and no patient had disease considered initially refractory to treatment. Coexistent infectious diseases were ruled out before starting the administration of GC and immunosuppressive agents.

The length of remission induction therapy ranged from 4 to 7 months and was replaced thereafter by remission maintenance therapy, with a gradually tapered dose of oral GC achieved with oral CYC in 4 patients, with MTX in 2 patients, with MMF in 2 patients, and with RTX in the remaining 14 patients. However, the dose of each agent in combination with GC was highly variable from patient to patient. CYC was given for a maximum period of 6 months, to reduce the risk of developing a malignant tumor (especially bladder cancer); it was then replaced by MTX or MMF or other immunosuppressive agents. Maintenance therapy was continued for 2–3 years or, for 3 patients with a shorter follow-up, until they were last seen.

Persistent ophthalmologic remission was achieved in 17 patients (77.3%) and persistent remission of extra-ocular manifestations in 14 patients (63.6%). Therapy withdrawal or poor therapeutic compliance was followed by one to three systemic relapses in 7 patients (31.8%), who were consistently re-treated with intravenous pulses of GC and RTX. Opportunistic and bacterial infections were diagnosed over the course of remission maintenance therapy in 3 and 4 patients, respectively, and were successfully treated with antibiotics prescribed on the basis of the antibiogram results. This group of 7 patients included 5 (22.7%) who suffered disease relapse with the same ophthalmologic manifestations detected at diagnosis (necrotizing scleritis in 2 patients and ulcerative keratitis, orbital disease, and optic neuropathy in 1 patient each). None of the relapses were exclusively ophthalmic. In the patient with necrotizing scleritis and a long follow-up (73 months) who developed bilateral cataracts, cataract extraction and intraocular lens implantation were performed. As shown in Table 3, at diagnosis no eye had BCVA of grade I, 23 eyes had grade II, 16 eyes grade III, and 5 eyes grade IV. At the last follow-up, BCVA was of grade I in 5 eyes, grade II in 26 eyes, grade III in 8 eyes, and grade IV in 5 eyes. Overall, 31 of 44 eyes (70.4%) were considered to have a good visual outcome (20/40 or better) at the last follow-up, but the BCVA of the 5 eyes with grade IV impairment remained unchanged throughout the follow-up.

Among the 7 patients with systemic manifestations at relapse, ENT and pulmonary lesions were diagnosed in 3 patients each, and renal involvement in 1 patient in whom the kidneys were unaffected at diagnosis. Three patients (13.6%) died: the causes of death were pneumonia from *Pneumocystis jirovecii* in a patient who had not received trimethoprim/sulfamethoxazole prophylaxis due to intolerance; severe relapse associated with sepsis of undefined etiology; and metastatic colon carcinoma, respectively (Table 3).

## Discussion

The experience acquired from large numbers of patients in single-center [23–25], polycenter [30–32], and nationwide [33–35] studies has clearly shown that GPA is a treacherous, necrotizing, granulomatous vasculitis of the small vessels, involving a single organ or characterized by multisystem involvement. Patients should be diagnosed as quickly as possible to avoid disease progression to severe or even life-threatening stages. The diagnosis is based on the association of clinical features, radiology imaging, ANCA testing, and, whenever possible, a tissue biopsy.

Renal, lung, and orbital tissue biopsies have a diagnostic sensitivity of 80–85% but, as they are invasive procedures, they were performed in less than half of the patients included in the large above-mentioned studies. Nasal and sinus biopsies, while more easily accessible, have a sensitivity of only ~ 60% [15, 36]. Although it is important to exclude a granulomatous infectious process, which can sometimes mimic GPA, the presence of granulomas, extravascular granulomatous inflammation, or giant cells on biopsies of pulmonary nodules, a pulmonary mass or cavitation, and/or pauci-immune glomerulonephritis on renal biopsy are the typical histopathological findings (Fig. 6) and can be highly informative in reaching a correct and possibly timely diagnosis [9, 18, 37]. In patients with disease restricted to an orbital mass or with no other accessible site, a section biopsy can be done via orbitotomy.

In our cohort of 63 patients, ocular manifestations were detected in 22 patients (34.9%) and similar percentages have been reported in the USA (30.1%) [38] and France (38.6%) [39]. However, a study in Spain [10] determined a remarkably lower incidence (13.3%) and studies in Russia (50%) [11] and the USA (58%) [40] a significantly higher incidence of the disease. In addition to ethnic, geographic, and environmental factors, these largely variable percentages may reflect a timely vs. late diagnosis and an occasional vs. a regular assessment of all patients by an ophthalmologist.

In GPA, ocular inflammation can occur with or without systemic manifestations of the disease [41]. The most common ocular features in our cohort were scleritis, without or with episcleritis or conjunctivitis, followed, in decreasing frequency, by retro-orbital pseudotumor and orbital mass, peripheral ulcerative keratitis, compressive neuropathy, retinal vasculitis, and uveal prolapse (Table 2). Although ophthalmic manifestations can be detected at any time during the course of the disease, in our patients combined ocular and systemic involvement at onset was slightly more frequent (40.9%) than systemic involvement followed at variable distance of time by the appearance of ocular lesion(s) (36.3%). In 4 patients (18.2%), ocular inflammation preceded the systemic manifestations. One patient (4.5%) was diagnosed with a sizeable orbital mass in the right eye that was responsible for proptosis, orbital discomfort, and conjunctival chemosis; he also had mild constitutional symptoms but no other organ involvement throughout his follow-up.

Overall, no correlation could be established between the type of ocular lesion and the location and extent of systemic manifestations. However, 15 of the 22 patients (68.2%) with ocular involvement had combined ocular and sino-nasal symptoms, a strikingly high frequency suggesting that ocular involvement is often the consequence of contiguous disease spread from the paranasal sinuses [42, 43]. Based on a comparison of BCVA at disease onset and at last follow-up, therapy resulted in a good visual outcome (20/40 or better) in 31 of 44 eyes (70.4%), although the BCVA of the 5 eyes with grade IV impairment remained unchanged throughout the follow-up (Table 3). The irreversible loss of visual acuity was ascribed to optic neuropathy in 2 eyes, retinal vasculitis in 2 eyes, and necrotizing scleritis in 1 eye. These findings are in keeping with those reported in several major series [12, 43, 44] and confirm that the visual prognosis is worse when the time to achieve remission is longer and the number of relapses is higher [42].

Worth mentioning is the possible relationship between respiratory tract involvement, ANCA positivity, and relapse. However, while previous studies suggested that ANCA persistence and lung involvement in patients who achieve therapy-induced remission are warning signs of relapse [43, 45, 46], no such correlation could be established in our patients. Of the 15 patients in our cohort who were ANCA-positive (14 c-ANCA and 1 p-ANCA) at diagnosis, 9 were still positive and 6 became negative at the end of remission maintenance. One to three relapses occurred in 7 patients, including 4 who were persistently ANCA-positive and 3 who were ANCA-negative or faintly ANCA positive. In addition, in 3 of the 7 patients with disease relapse, the upper and/or lower respiratory tract were unaffected at the time of remission induction therapy and remained unaffected during relapse(s). Nonetheless, given the relatively small number of patients in our cohort, definite conclusions cannot be reached as to whether persistent ANCA positivity and lung involvement should be considered as predictors of treatment resistance or relapse.

The treatment of GPA requires a personalized and frequently complex schedule that involves multiple combinations of drugs for long periods of time, depending on the level of disease severity at presentation and throughout its course. The objectives of remission induction therapy are to curb disease progression and the consequent multi-organ involvement and to subsequently prevent disease recurrence. For many years, the combination of GC and the alkylating agent CYC was considered the gold standard for the induction of remission [20, 23, 47], and this regimen was often continued unchanged for remission maintenance. However, despite its undoubted effectiveness, it is important to reduce the duration of CYC treatment, given its adverse consequences, which include bone marrow hypoplasia, infertility, and hemorrhagic cystitis possibly progressing to bladder cancer. The cumulative CYC dose above which the risk of bladder cancer sharply rises reportedly ranges from > 20 g to > 100 g [48]. Treatment with Mesna, regularly employed in our patients, can minimize but does not eliminate this risk.

Based on these considerations, EULAR [20] proposed that, for remission maintenance, CYC should be replaced by a combination of GC and other, less toxic immunosuppressive agents such as azathioprine [49], MTX [47], or MMF [50]. However, even after 2 years of remission maintenance therapy with these agents, the rate of relapse is relatively high [47]. In our cohort, in addition to GC, the immunosuppressive agents employed in the remission induction phase were CYC in 11 patients, RTX in 10 patients (including 5 who had previously received and were unresponsive to CYC), MTX in 3 patients, and MMF in 3 patients. In the remission maintenance phase, in step with a gradual tapering of GC, RTX was the drug most frequently employed (14 patients), followed by CYC (4 patients) and MTX and MMF (2 patients each).

Introduction of the B-cell-depleting monoclonal antibody RTX was a milestone in the therapy of AAV, as clearly shown in network studies. RTX was found to be not inferior to daily CYC for remission induction and was even more effective in patients with relapsing disease [51]. In a multicenter randomized study, a single course of RTX was as effective as continuous conventional immunosuppressive therapy with CYC followed by azathioprine for remission induction and maintenance over the course of 18 months [52]. In addition, a significantly higher proportion of patients achieved sustained remission at month 28 with RTX than with azathioprine [53] and the prolongation of RTX in the maintenance regimen for 36 months resulted in a significantly higher percentage of relapse-free survival than obtained in a control group receiving placebo (96 and 74%, respectively) [54]. Thus, compared with the most common immunosuppressive agents, RTX is safer and more effective [55] and its long-term administration should accordingly be considered the standard of care for AAV [54, 56].

Consistent with our results in the treatment of ophthalmologic involvement in GPA, RTX, as an induction and maintenance treatment, was shown to be just as effective as CYC in patients with scleritis [57], was comparable to CYC in terms of safety and efficacy in those with scleritis and uveitis [58] and was able to achieve remission in > 87% of a small cohort of patients with refractory necrotizing scleritis [59]. RTX likewise resulted in a successful outcome in patients who were retreated after relapse [60]. RTX-induced remission was also achieved in patients with localized and generalized ocular GPA [55]. In patients with an orbital mass, CYC has been frequently used as first-line therapy followed by RTX as the maintenance regimen, with a complete remission achieved in a minority of patients [42, 60]. Given the great heterogeneity of the clinical manifestations and the complexity of the therapeutic combinations, multi-specialty collaboration is strongly advised to achieve the best therapeutic results.

The strengths of the present study are: 1) the homogeneous collection of data as a result of the multi-year collaboration between the tertiary eye-care and clinical immunology centers of the same university hospital; 2) the clinical and ophthalmologic assessments made by the same internists and the same ophthalmologists, thus reducing the risk of unwanted variability; 3) the contribution to the design of the study and the interpretation of its results by the Department of Ocular Pathology, Sankara Nethralaya, Chennai, and the Department of Ophthalmology, University of Mainz; and 4) the length of follow-up after the onset of ocular involvement (median 51 months, range 21–84 months), which exceeded 5 years in 42% of the study patients. However, the following limitations must be noted as well: 1) the retrospective nature of the study; 2) the relatively small cohort of 63 patients (22 of them with ocular manifestations), compared to the much larger number of patients enrolled in polycenter or retrospective population-based cohort studies [10–12, 23, 24, 51]; 3) the ocular and extra-ocular manifestations systematically assessed at diagnosis but which were more fragmentary in the subsequent controls carried out at irregular intervals; and 4) the heterogeneity of the drugs and their combinations employed in both remission induction and remission maintenance therapies. Although inevitable, given the long-time frame (almost 15 years) during which the patients were diagnosed, the heterogeneous treatment hindered reliable comparisons of the clinical responses among patients.

Based on the data obtained in this cohort, we recommend an ophthalmological examination for all GPA patients to allow the early recognition of ocular injury and thus the prevention of the often severe consequences of a late diagnosis. We also highlight the recent advances in the therapeutic landscape marked by the introduction of B-cell-depleting monoclonal antibodies and other biologic agents, which have remarkably improved the outcome of GPA patients, including those with relapsing or refractory disease.

## Data Availability

The data that support the findings of this study are available on request from the corresponding author. The data are not publicly available due to their containing information that could compromise the privacy of research participants.

## Abbreviations

AAV: ANCA-associated vasculitides
ANCA: anti-neutrophil cytoplasmic antibody
BCVA: best-corrected visual acuity
BVASv3: Birmingham Vasculitis Activity Score version 3
CT: computed tomography
CYC: cyclophosphamide
ENT: ear/nose/throat
EULAR: European League Against Rheumatism
FDG-PET/CT: ^18^F-fluorodeoxyglucose positron emission tomography/computed tomography
GC: glucocorticoids
GPA: granulomatosis with polyangiitis
IOP: intraocular pressure
MMF: mycophenolate mofetil
MPO: myeloperoxidase
MTX: methotrexate
PET: positron emission tomography
PR3: proteinase-3
PTX3: pentraxin-3
RTX: rituximab
